# Pediatric stroke in inborn errors of metabolism: clinical characteristics, neuroimaging features, and short-term outcomes

**DOI:** 10.3389/fneur.2026.1889684

**Published:** 2026-09-03

**Authors:** Javeria Raza Alvi, Areeba Wasim, Saher Gul Ahdi, Tipu Sultan, Muhammad Zia-ur-Rehman, Khalid Al-Thihli, Fathiya Al Murshedi, Amna Al Futaisi

**Affiliations:** 1 Department of Pediatric Neurology, University of Child Health Sciences, The Children’s Hospital, Lahore, Pakistan; 2 Child Health Department, Pediatric Neurology Division, Sultan Qaboos University Hospital, Muscat, Oman; 3 Genetics Department, Sultan Qaboos University Hospital, University Medical City, Muscat, Oman; 4 Department of Child Health, College of Medicine and Health Sciences, Sultan Qaboos University, Muscat, Oman

**Keywords:** acute neurological deficits, functional outcome, genetics, inherited metabolic disorders, neuroimaging, stroke-like episodes

## Abstract

**Introduction:**

Pediatric stroke has a broad etiological spectrum, with inborn errors of metabolism (IEM) increasingly recognized as an important and potentially treatable cause. This study aimed to characterize the clinical, radiological, and electroencephalographic features of IEM-related pediatric stroke and to evaluate short-term functional outcomes.

**Methods:**

This prospective cohort study was conducted at The Children’s Hospital Lahore, Pakistan, and Sultan Qaboos University Hospital, Muscat, Oman (January 2024–December 2025). Children with confirmed stroke secondary to IEM were enrolled. Functional outcome was assessed at 3 months using the modified Rankin Scale (mRS), with scores 0–3 defined as favorable and 4–6 as unfavorable.

**Results:**

Forty-four patients were included (59.1% male), with a mean age of 4.6 ± 4.2 years. Focal neurological deficits and encephalopathy were the most frequent presentations. Mitochondrial disorders and homocystinuria were the leading etiologies. MRI demonstrated heterogeneous patterns, most commonly focal ischemic lesions and brainstem–striatal involvement. Management included mitochondrial cocktails, vitamin therapy, and targeted metabolic treatments. Poorer outcomes (mRS 4–6) were associated with generalized epileptiform discharges with diffuse slowing on EEG, whereas favorable outcomes correlated with focal epileptiform discharges or normal EEG findings (*p* = 0.00018). Outcomes also varied by etiology, with homocystinuria and mitochondrial complex I deficiency associated with better recovery and Leigh disease and glutaric aciduria with worse prognosis (*p* = 0.041).

**Conclusion:**

IEM represent an important and potentially treatable cause of pediatric stroke, particularly in populations with high consanguinity. Recognition of characteristic clinical features, distinctive MRI patterns, and supportive EEG findings can facilitate early diagnosis. Prompt metabolic and genetic evaluation is essential to guide targeted therapy and improve outcomes.

## 1 Introduction

Pediatric stroke has garnered wider recognition over the past few years as a significant cause of childhood morbidity, with a significant increase in incidence of more than 33 million affected individuals per year (1). The etiology of stroke in children is diverse. In particular, stroke due to IEM, while rare, is increasingly recognized as a significant inherited etiology. In stroke due to IEM, a cascade of stress-induced metabolic derangements precipitates the rapid onset of neurological deficits (2). Deficits may vary from motor, verbal, and cognitive impairments to acute encephalopathy with or without associated multisystem involvement. Specifically, Metabolic stroke/Stroke-like episodes is defined as a rapidly developing focal or global neurological deficit caused by acute metabolic dysfunction rather than from a vascular occlusion (2).

The presentation of stroke due to IEM varies widely, even among inherited metabolic disorders, with distinct clinical features and neuroimaging patterns. Metabolic stroke/Stroke-like episodes are classically observed in mitochondrial disorders such as mitochondrial encephalopathy, lactic acidosis, and stroke (MELAS), Leigh disease, and congenital glycosylation disorders involving focal deficits and cranial neuropathies (specifically, ophthalmoplegia). Here, defective mitochondrial proliferation of small arteries and arterioles leads to stroke-like episodes with discrete ischemic lesions on neuroimaging that are not confined to a specific vascular territory (3). Moreover, bilateral striatal necrosis and brainstem lesions can develop from mitochondrial damage, as seen in Leigh disease (4, 5). Classical arterial (territorial) stroke is typically associated with homocystinuria (6), where elevated homocysteine levels lead to coagulopathy, hypercholesterolemia, and endothelial damage, causing thromboembolism. Metabolic stroke can also present with diffuse and extensive white matter lesions, as evident in Fabry’s disease, which features multisystem cardiac and renal involvement. Organic acidemias are a group of IEMs in which episodes of metabolic decompensation often result in focal neurological deficits that preferentially involve the basal ganglia, although cortical and subcortical white matter can also be affected (7).

The advent of advanced metabolic and genetic testing has enabled the earlier identification and prompt treatment of many inherited metabolic disorders. Although the diagnosis and management of these rare conditions have progressed significantly, substantial gaps remain, underscoring the need for continued research and development.

The primary objective of this study was to identify the key clinical and radiological features to help elucidate the underlying etiology of stroke due to IEM in children. The secondary objective was to evaluate short-term functional outcomes at follow-up and examine their association with the specific underlying metabolic disorder.

## 2 Methods

### 2.1 Study design and setting

This descriptive, prospective study was conducted at the Department of Pediatric Neurology, The Children’s Hospital and The University of Child Health Sciences, Lahore, Pakistan, and Department of Child Health, Sultan Qaboos University Hospital, Muscat, Oman, from January 2024 to December 2025. This study was approved by the respective institutional review boards (IRB No.667/CH-UCHS, SQU-EC/138/2025, MREC # 3570, respectively). Sample size was determined to be 44, based on a reported stroke incidence of 2.9 per 100,000 in children less than 18 years of age, with a 95% confidence interval and 5% margin of error. All calculations were made using openepi.net.

Consecutive patients aged 0–18 years with a confirmed diagnosis of an inborn error of metabolism (IEM) who developed a stroke syndrome during the study period were included. Eligible patients comprised those with vascular stroke (including arterial ischemic stroke, cerebral venous sinus thrombosis, and intracerebral hemorrhage) as well as metabolic stroke-like episodes. The diagnosis of IEM was established by characteristic biochemical abnormalities on metabolic investigations (including plasma ammonia, lactate, plasma amino acids, urine organic acids/chromatography, and plasma homocysteine levels) and/or molecular genetic testing. Stroke and stroke-like episodes were confirmed by neuroimaging (MRI and/or CT) along with the clinical presentation.

Patients with stroke attributable to causes other than an IEM were excluded. These included cardioembolic disease, extracranial or intracranial arterial dissection, hematological disorders, trauma, central nervous system infection, acquired inflammatory or demyelinating disorders, brain tumors, or cryptogenic stroke. Patients with suspected but unconfirmed IEM, those without neuroimaging evidence consistent with stroke or stroke-like episodes, and those with incomplete clinical or diagnostic data were also excluded.

The modified Rankin Scale (mRS) was used to assess functional outcome (8). It is a 6-point disability scale ranging from 0–6, where 0 indicates no symptoms, 1 no significant disability, 2 slight disability, 3 moderate disability, 4 moderately severe disability, 5 severe disability and 6 dead. We categorized the outcomes with grades 0–3 indicating better outcomes and 4–6 indicating worse outcomes.

### 2.2 Data collection

Data collection was performed using a predesigned form after informed consent was obtained from the pediatric patient’s caregiver. The demographic details; family history; developmental milestones; detailed birth history; and clinical manifestations comprising the onset of stroke, localization of the lesion, associated features like seizures, their age of onset, and type of epilepsy as per International League Against Epilepsy classification were documented (9). Various neurological symptoms, such as neuroregression, ataxia, dystonia, neuropathy, and myopathy, were documented as related to metabolic errors, which can present with multiple symptoms. Detailed systemic inquiry was conducted, considering a multisystem basis of disease (e.g., cardiac, visual, renal, and endocrine abnormalities), as indicated. Detailed testing of metabolic parameters (ammonia, lactate, urinary chromatogram, and plasma amino acids), homocysteine levels, video electroencephalography (EEG), magnetic resonance imaging (MRI), and fundus examination results were documented. Whole-exome sequencing was performed for genetic testing. Neurological status at the outset of the disease and after 3 months was evaluated using mRS.

### 2.3 Data analysis

The data were analyzed using BM SPSS Statistics for Windows, Version 26.0 (IBM Corp., Armonk, NY, United States). Frequency and percentage were calculated for qualitative variables, including sex, parent consanguinity, seizures, stroke, ataxia, neuro-regression, neuropathy, myopathy, visual and associated problems, and EEG review and neuroimaging data. Mean and standard deviation were calculated for quantitative variables such as age, time to diagnosis, and mRS scores. Chi-square and Fisher’s exact tests were used for poststratification analysis. Statistical significance was set at <0.05.

## 3 Results

The study included 44 patients with stroke due to IEM, of whom (26/44; 59.1%) were male and the mean age was 4.6 ± 4.2 years (Table 1). Consanguinity was present in (37/44; 84.1%) patients, and (13/44; 29.5%) had a sibling with a similar history. All 44 patients (100%) exhibited acute neurological deficits at presentation or during the disease course, most often right-sided focal deficit (21/44; 47.7%). The mean age at stroke onset was 3.8 ± 4.1 years. Cranial nerve involvement was noted in 16 patients (36.4%), manifesting as ophthalmoplegia, upper motor neuron-type facial palsy, or bulbar palsy.

**Table 1:** Distribution of study participants by baseline and clinical characteristics.

Characteristics	Frequency *n* (%)
Sex	
Male	26 (59.1%)
Female	18 (40.9%)
Age in groups	
1 month to 2 years	15 (34.1%)
>2–5 years	12 (27.3%)
>5–10 years	10 (22.7%)
>10–15 years	7 (15.9%)
Clinical Presentation at outset	
Isolated stroke	13 (29.5%)
Stroke and seizures	4 (9.1%)
Stroke and encephalopathy	1 (2.3%)
Seizures	6 (13.6)
Acute encephalopathy	8 (18.2%)
Seizures and encephalopathy	6 (13.6%)
Headache	1 (2.3%)
Quadriparesis	1 (2.3%)
Vomiting	1 (2.3%)
Ataxia	1 (2.3%)
Dystonia	2 (4.5%)
Lateralizing weakness	
Left	16 (36.4%)
Right	21 (47.7%)
Bilateral	7 (15.9%)
Recurrence of stroke	
Yes	12 (27.3%)
No	32 (72.7%)
Associated neuro-ophthalmological features	
Dystonia	16 (36.4%)
Myopathy	3 (6.8%)
Neuropathy	3 (6.8%)
Retinitis Pigmentosa	4 (9.1%)
Optic disc pallor	5 (11.4%)
Subluxation of lens	1 (2.3%)
Glaucoma	1 (2.3%)
Visual field dysfunction	4 (9.1%)
Modified Rankin Score at outset of disease	4.14 ± 0.79

Seizures were observed in 34 patients (77.3%), 16 (47%) of whom had seizures as a presenting symptom along with focal deficit, while 18 (53%) developed them during the course of illness; the most common seizure type was generalized seizure (13/34, 38.2%) including tonic clonic in (8/13, 61.5%) and myoclonic in (5/13, 38.5%), followed by focal impaired consciousness seizure (12/34, 35.3%), focal to bilateral tonic–clonic seizure (8/34, 23.5%), and seizure of unknown onset (1/34, 2.9%).

**Figure 1:**
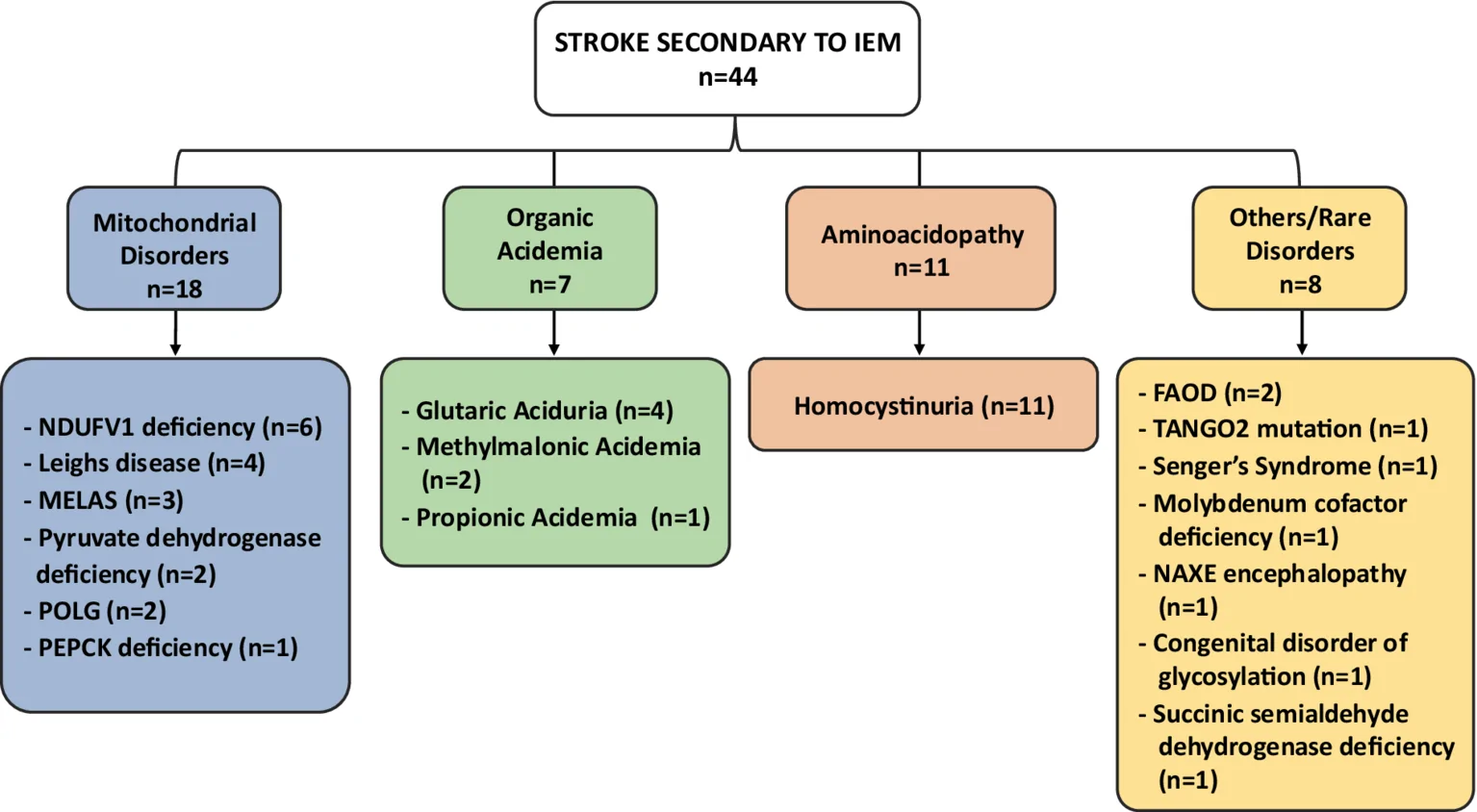
Breakdown of etiological factors. IEM, Inborn error of metabolism; NDUFVI, NAH dehydrogenase flavoprotein I; MELAS, Mitochondrial encephalopathy with lactic acidosis and stroke; POLG, Polymerase gamma; PEPCK, Phosphoenolpyruvate carboxykinase; FAOD, Fatty acid oxidation defect; TANG02, Transport and Golgi organization.

Status epilepticus occurred in 18 (40.9%) patients. The mean time to reach a final diagnosis of metabolic etiology was 2.3 ± 1.0 years. The most common etiological group was mitochondrial disorders, accounting for 18 (40.9%) cases, with breakdown of etiology presented in Figure 1.

The adjunct neurological features are mentioned in Table 1. Neuropathy was observed in patients with Leigh disease (*n* = 2), NAXE encephalopathy (*n* = 1), and homocystinuria (*n* = 1), while myopathy occurred in Leigh disease (*n* = 2) and propionic acidemia (*n* = 1), and dystonia was predominantly associated with mitochondrial disorders and organic acidemias.

Ophthalmological manifestations included optic disc pallor in Leigh disease (*n* = 2), NDUFV1 complex I deficiency (*n* = 1), and glutaric aciduria type 1 (*n* = 2); Retinitis pigmentosa in mitochondrial disorders (*n* = 3); lens subluxation in homocystinuria (*n* = 2) and visual pathway dysfunction in mitochondrial disorders (*n* = 3) and molybdenum cofactor deficiency (*n* = 1).

**Figure 2:**
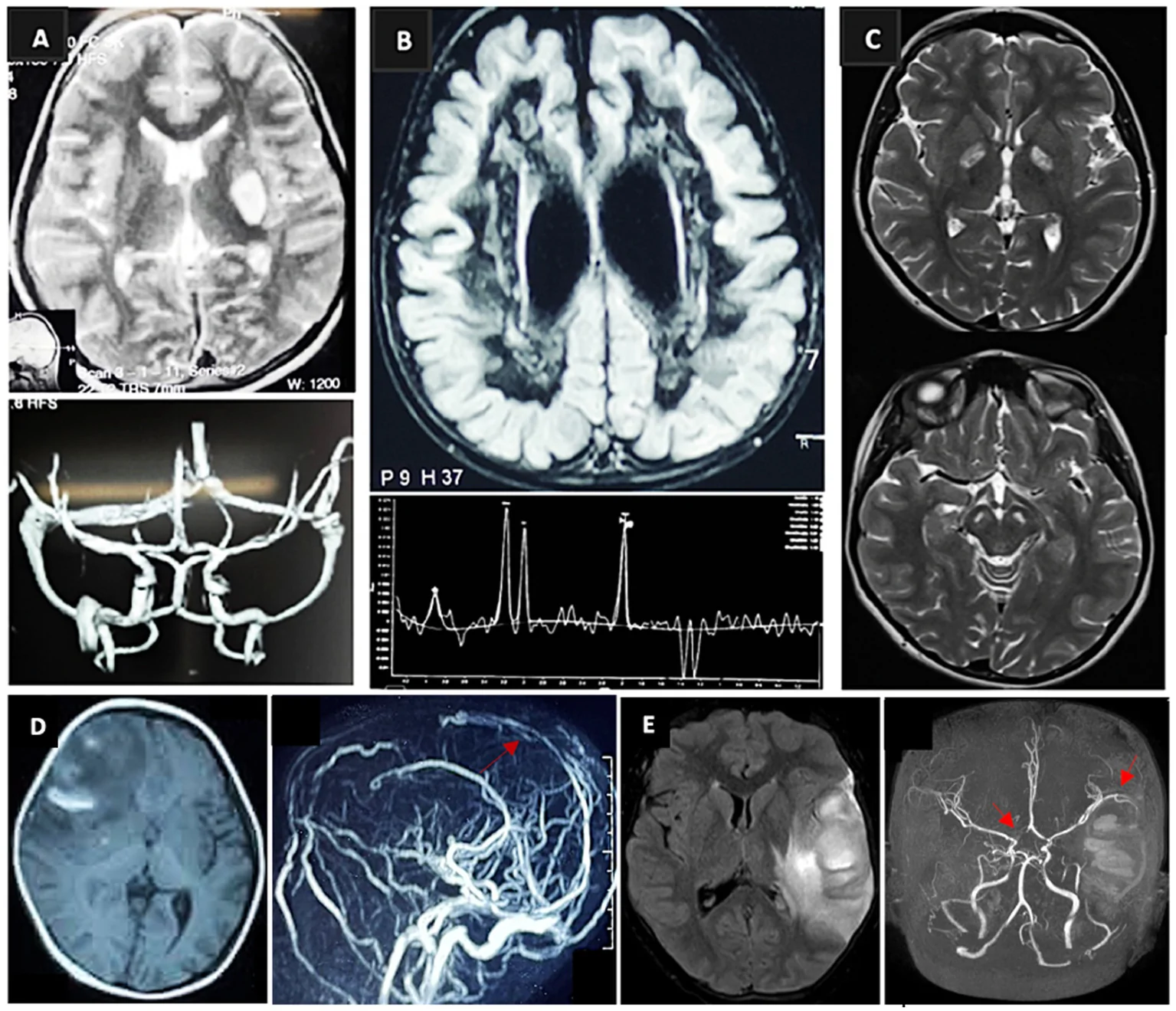
Spectrum of neuroimaging findings with various metabolic errors.\**(A)** Case of Senger’s Syndrome; upper panel: Axial T2-weighted MRI shows left hyperintense signal in the putamen. Lower panel: MR angiography (MRA) demonstrates normal vascular imaging.\**(B)** Case of Mitochondrial Complex 1 deficiency; Axial FLAIR MRI shows bilateral periventricular and subcortical white matter hyperintensities. Lower panel: MR spectroscopy reveals a lactate doublet peak.\**(C)** Case of Pyruvate Dehydrogenase Alpha Deficiency; Axial T2-weighted MRI reveals symmetrical hyperintensities in globus pallidus (top) and in dorsal brainstem (bottom).\**(D)** Case of homocystinuria; Axial T1W post-contrast MRI (left) shows hemorrhagic foci with hypointense area in the right parietal region causing contralateral shift. Sagittal MR venography (right) displays narrowing of superior sagittal sinus and non-visualization in its anterior part.\**(E)** Case of homocystinuria; Axial DWI showing diffusion restriction in the left parieto-temporal region in a wedge-shaped pattern (left). MR angiography (right) narrowing of intracavernosal part of right internal carotid artery and right anterior cerebral artery along with paucity of branches of left middle cerebral artery.

Regarding the metabolic workup, 15 (34.1%) patients had metabolic acidosis, 6 (13.6%) had hyperammonemia, 23 (52.3%) had lactic acidosis, and 11 (25%) had hyperhomocysteinemia. On urinary gas chromatography, 27 (61.4%) patients had insignificant findings, while the rest showed peaks of dicarboxylic acid (*n* = 3), methylmalonic acid (*n* = 2), glutaric acid (*n* = 2), hydroxybutyric acid (*n* = 2), glutaconic acid (*n* = 2), low glycine (*n* = 1), propionic acid (*n* = 1), and oxoproline (*n* = 1). The diverse spectrum of EEG and neuroimaging features observed are shown in Table 2 and Figure 2.

**Table 2:** Spectrum of EEG and neuroimaging findings.

Characteristics	Frequency *n* (%)
EEG FINDINGS	*n* = 38
Focal epileptiform discharges	9 (20.5%)
Multifocal epileptiform discharges	6 (13.6%)
Generalized epileptiform discharges	4 (9.1%)
Diffuse slowing	10 (22.7%)
Modified hypsarrhythmia	1 (2.3%)
Asymmetry with bilateral discharges	1 (2.3%)
Normal	7 (15.9%)
NEUROIMAGING FINDINGS	*n* = 44
*Distribution of lesions*	
Unilateral	12 (27.3%)
Bilateral	32 (72.7%)
*Neuroradiology findings*	
Multifocal diffusion restriction not confined to a vascular territory	8 (18.2%)
Diffusion restriction confined to single vascular territory	4 (9.1%)
Bilateral striatal necrosis	4 (9.1%)
Bilateral symmetrical periventricular white matter hyperintensities with cavitation	4 (9.1%)
Bilateral intracranial arterial stenosis on MR angiography	3 (6.8%)
Sulcal hyperintensities with absent venous flow on MR venography	6 (13.6%)
Diffusion restriction in globus pallidi and crura of midbrain	1 (2.3%)
Unilateral striatal ischemia	2 (4.5%)
Bilateral striatal necrosis with subdural hemorrhage	4 (9.1%)
Diffuse hemispheric involvement with associated cerebellar lesion	1 (2.3%)
Agenesis of corpus callosum with bilateral symmetrical white matter hyperintensities and deep nuclei involvement	1 (2.3%)

EEG, Electroencephalography; MRI, Magnetic resonance imaging; MRA, Magnetic resonance angiography; MRV, Magnetic resonance venography.

Patients were treated with a mitochondrial cocktail of carnitine, co-enzyme Q10 in 18 (40.9%) patients: IV arginine +/− citrulline, vitamins, including pyridoxine and cobalamin in 13 (29.4%); ketogenic diet in 1 (2.3%); and immune therapy, including intravenous immunoglobulin and steroids in 1 (2.3%) case initially diagnosed with brainstem encephalitis that later turned out to be NAXE encephalopathy. These treatments were administered alongside infection management and neuroprotective measures. Anti-seizure medication (ASM) use included one ASM in 9 patients (20.5%), two ASMs in 7 (15.9%), three ASMs in 11 (25.0%), and more than three ASMs in 7 (15.9%). The mRS score at presentation was 4.1 ± 0.79, which improved to 2.9 ± 1.5 at the 3-month follow-up. Stroke recurred in 12 (27.3%) cases with homocystinuria (77%) or mitochondrial disorders (33%).

**Figure 3:**
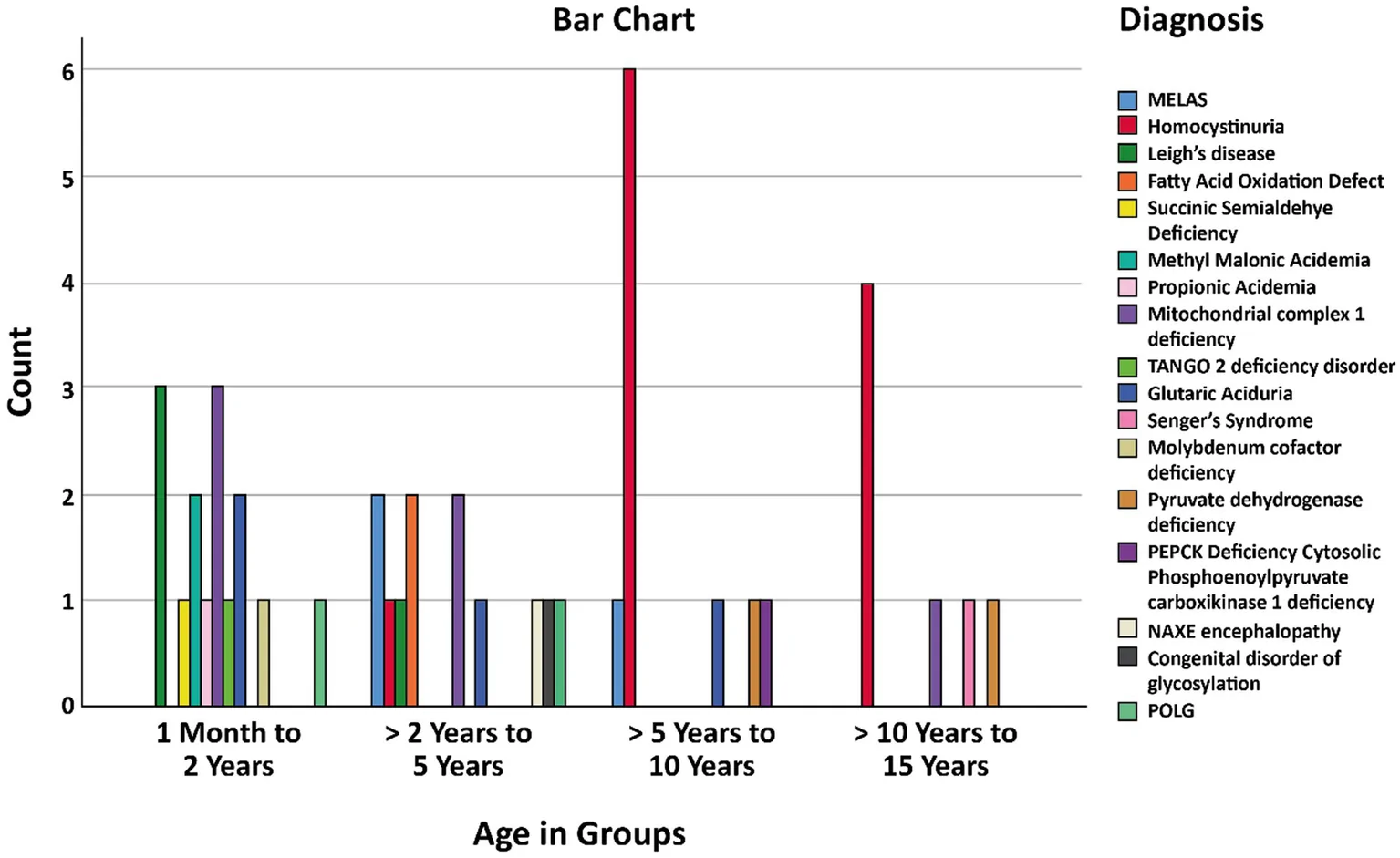
Age-wise distribution across different metabolic stroke.

Etiologies associated with better outcomes at 3 months’ follow-up (mRS 0–3) included homocystinuria and mitochondrial complex 1 deficiency, while Leigh disease, GA1, propionic aciduria, and NAXE encephalopathy were associated with worse outcomes (mRS 4–6) indicating statistically significant correlation between etiology and mRS outcome (*p* = 0.041). A significant association was observed between EEG pattern and functional outcome, with generalized epileptiform discharges and diffuse slowing associated with moderately severe to severe disability (mRS 4–6), while focal epileptiform discharges and normal EEG findings were more common among patients with mRS 0–3 (*p* = 0.00018). (Table 3). A significant association was observed between age in group and etiological factor (*p* = 0.015, Fisher’s exact test), suggesting that certain metabolic disorders that cause stroke are more likely to present at specific ages (Figure 3). However, sex (*p* = 0.060), age in groups (*p* = 0.397), lateralizing weakness (*p* = 0.150), seizure type (*p* = 0.512), MRI findings (*p* = 0.680), and high lactate levels (*p* = 0.119) were not significantly associated with mRS outcomes.

**Table 3:** Correlation of sex, age, etiology, and EEG features with mRS score at follow-up.

Characteristics	mRS 0–3 (*n* = 27)	mRS 4–6 (*n* = 17)	*p*-value
Sex			
Male	13 (48.1%)	13 (76.5%)	0.060^a^
Female	14 (51.9%)	4 (23.5%)	
Age in groups			
1 month to 2 years	8 (29.6%_	7 (41.3%)	0.391^a^
>2 months to 5 years	6 (22.2%)	6 (35.3%)	
>5 years to 10 years	7 (26.0%)	3 (17.6%)	
>10 years to 15 years	6 (22.2%)	1 (5.8%)	
Etiology			
MELAS	1 (3.7%)	2 (11.8%)	0.041^b^
Mitochondrial complex 1 deficiency	6 (22.3%)	2 (11.8%)	
Leigh’s disease	0 (0.0%)	4 (23.5%)	
Pyruvate dehydrogenase deficiency	2 (7.4%)	0 (0.0%)	
PEPCK deficiency	1 (3.7%)	0 (0.0%)	
Senger’s syndrome	1 (3.7%)	0 (0.0%)	
Homocystinuria	8 (29.6%)	3 (17.6%)	
Succinic semialdehyde deficiency	1 (3.7%)	0 (0.0%)	
Methylmalonic acidemia	2 (7.4%)	0 (0.0%)	
Glutaric aciduria	1 (3.7%)	3 (17.6%)	
Propionic acidemia	0 (0.0%)	1 (5.9%)	
Fatty acid oxidation defect	2 (7.4%)	0 (0.0%)	
TANGO 2 deficiency	1 (3.7%)	0 (0.0%)	
*NAXE*-associated encephalopathy	0 (0.0%)	1 (5.9%)	
Congenital disorder of glycosylation	0 (0.0%)	1 (5.9%)	
Molybdenum cofactor deficiency	1 (3.7%)	0 (0.0%)	
EEG Findings			
Focal epileptiform discharges	6 (22.3%)	3 (17.6%)	0.00018^a^
Multifocal epileptiform discharges	4 (14.8%)	2 (11.8%)	
Generalized epileptiform discharges	0 (0.0%)	4 (23.5%)	
Diffuse slowing	2 (7.4%)	8 (47.1%)	
Modified hypsarrhythmia	1 (3.7%)	0 (0.0%)	
Asymmetry with bilateral discharges	1 (3.7%)	0 (0.0%)	
Normal	7 (25.8%)	0 (0.0%)	
Not performed (No seizures)	6 (22.3%)	0 (0.0%)	

^a^Chi-square test, ^b^Fischer exact test. EEG, Electroencephalography; mRS, modified Rankin Scale score; MELAS, Mitochondrial encephalopathy, lactic acidosis and stroke; PEPCK, Phosphoenolpyruvate carboxykinase; TANGO2, Transport and Golgi organization 2.

## 4 Discussion

Pediatric stroke due to IEM is a challenging entity because of its atypical presentation and overlapping features. This study of 44 patients highlights the clinical complexity, diagnostic delays, and diverse outcomes associated with different IEMs, with important implications for early identification and tailored management. The high consanguinity rate (84.1%) observed in this study aligns with the findings of Afzal et al., (10), who reported a 30-fold increase in the incidence of IEM among Pakistanis born to consanguineous unions. The finding also highlights the autosomal recessive pattern of inheritance, which is typical of most IEMs. The peak number of strokes was clustered in the toddler age group, consistent with previous reports (11). This reflects the overlap between early onset clinical features and increased vulnerability of infants to metabolic decompensation.

Będkowska et al., (12) reported stroke-like episodes (SLE) in IEM, particularly with MELAS, Leigh disease, and congenital disorder of glycosylation involving focal deficits as well as cranial neuropathies, specifically ophthalmoplegia, aligning with our study. Our cohort also included rarer etiologies, such as succinic semialdehyde dehydrogenase (SSADH) and molybdenum cofactor (MOCS) deficiency, NAXE encephalopathy, and Senger syndrome. SSADH deficiency typically presents with infantile hypotonia, developmental delay, seizures, and movement disorders (13). One patient in our series initially experienced normal development and eventually presented with stroke as the initial manifestation. A significant family history of seizures and developmental delay prompted an expanded metabolic evaluation, leading to the diagnosis.

Similarly, MOCS deficiency typically presents with global developmental delay and refractory seizures. However, our patient presented at 3 months with acute encephalopathy, recurrent seizures, and a stroke-like episode. Laboratory evaluation showed undetectable uric acid levels, while neuroimaging demonstrated a hemispheric infarct with normal angiography. Whole-exome sequencing identified a homozygous MOCS2 variant (NM_176806.3:c-10_13del). At follow-up, the patient had persistent axial and appendicular hypotonia, inconsistent visual fixation, and an mRS score of 4. A similar stroke-like presentation was reported by Markose et al., (14) although their patient presented at 2 years of age and had a more favorable outcome. Several rare causes of stroke were identified. A patient with NAXE encephalopathy presented at 20 months with acute encephalopathy, quadriparesis, ptosis, ophthalmoplegia, and stroke involving the bilateral thalami, brainstem, and cerebellum. Although NAXE encephalopathy most commonly manifests as progressive epileptic encephalopathy, quadriparesis and cranial neuropathies are exceedingly rare (15, 16). An 11-year-old girl with Sengers syndrome presented with cardiomyopathy, cataracts, and a stroke-like episode causing right-sided pure motor hemiparesis. Elevated serum lactate and a homozygous AGK variant confirmed the diagnosis. While the core phenotype includes cataracts, cardiomyopathy, lactic acidosis, and skeletal myopathy, stroke-like episodes have not previously been reported in Sengers syndrome (17).

Seizures were also a prominent feature, affecting 77.3% of patients. Furthermore, approximately two-thirds of patients developed status epilepticus (47%), which was most commonly seen in MELAS, Leigh disease, and mitochondrial complex 1 deficiency but less commonly in homocystinuria. This observation is consistent with existing literature, which found an association between focal or generalized seizures and stroke-like episodes in mitochondrial disorders, likely reflecting underlying metabolic instability and increased neuronal excitability (18). Although no disease-specific EEG pattern was identified in our cohort, continuous EEG monitoring remains essential. Even in the absence of clinically apparent seizures, EEG can detect subclinical epileptiform activity that might otherwise go unrecognized.

Recurrence of stroke was observed in 27.3% of our cases, with a notable proportion of homocystinuria as a cause (66.6%). Tabarki et al., (11) reported that approximately 32% of patients with untreated cystathionine B synthase deficiency experience thromboembolic events, which was also observed in our cohort. Recurrence of pediatric stroke related to metabolic disorders is most frequently associated with methylenetetrahydrofolate reductase (MTHFR) deficiency or ornithine transcarbamylase (OTC) deficiency (19, 20). However, this pattern was not observed in our cohort. This discrepancy could be due to high consanguineous marriages, which increases the burden of autosomal recessive disorders. Moreover, stroke recurrences in MTHFR and OTC deficiencies may be underrecognized or misclassified due to their multifactorial presentation.

In our cohort, dystonia was observed in 36.4% of patients, consistent with its established association with metabolic disorders affecting the basal ganglia, particularly organic acidemias and mitochondrial diseases (21). Myopathy was diagnosed in one patient with a mitochondrial disorder, a well-documented manifestation in literature (22), as well as in a patient with propionic acidemia. Although propionic acidemia classically presents with seizures, movement disorders, or hypotonia (23), emerging evidence indicates it may also manifest as a form of metabolic myopathy extending beyond the traditional mitochondrial spectrum (24). Peripheral neuropathy is a well-established feature in mitochondrial cytopathies, as observed in one of our patients (25). However, we also observed neuropathy manifest in homocystinuria and NAXE encephalopathy, an association rarely reported in the context of metabolic stroke (16, 26). Interestingly, while approximately 45.4% of patients had metabolic stroke, none exhibited movement disorders or neuromuscular signs. This underscores the need for a thorough metabolic work-up in all pediatric stroke cases.

Ophthalmological manifestations were observed across several metabolic etiologies, including mitochondrial disorders, organic acidemias, homocystinuria, and MOCS deficiency, and served as important clinical clues of an underlying metabolic cause (10). Notably, MOCS deficiency has only rarely been associated with stroke-like episodes and encephalopathy, consistent with our case. Furthermore, ocular abnormalities, such as ectopia lentis and glaucoma, have been reported in MOCS deficiency, further highlighting its multisystem involvement (14, 27).

Neuroimaging revealed a broad, etiology-specific spectrum of abnormalities. Brainstem and striatal involvement were extensive in patients with MELAS, Leigh disease, and pyruvate dehydrogenase deficiency. Bilateral striatal lesions with restricted diffusion during acute episodes were most frequently associated with organic acidemias, including methylmalonic acidemia, propionic acidemia, and glutaric aciduria. Mitochondrial complex I deficiency demonstrated bilateral symmetrical white matter hyperintensities with cystic vacuolation (Figure 2B). Homocystinuria exhibited variable imaging findings, with sulcal hyperintensities and venous thrombosis observed in the majority of cases, followed by either unilateral arterial narrowing (Figures 2D,E) or bilateral intracranial arterial stenosis with collaterals in one patient on MR angiography. While these radiological patterns align with previous studies and provide important diagnostic clues (28, 29), several novel observations emerged in our cohort including thalamic, brainstem, and cerebellar lesions in NAXE encephalopathy, hemispheric infarct in MOCS2 deficiency, and unilateral striatal lesions in SSADH deficiency and Senger syndrome.

The mean mRS score at presentation was 4.1 ± 0.79, which improved to 2.9 ± 1.5 at the 3-month follow-up. In our cohort, children with mitochondrial complex I deficiency and homocystinuria demonstrated comparatively better outcomes, consistent with prior findings by Demartini et al., (30). In contrast, patients with Leigh disease, GA1, propionic aciduria, and NAXE encephalopathy showed poorer outcomes. These findings suggest that prognosis is strongly influenced by the underlying metabolic etiology.

Timely diagnosis is critical, as stroke due to IEM may mimic encephalitis, demyelinating disorders, or focal cerebral arteriopathies, delaying appropriate management. Early recognition of characteristic clinical and radiological features enables prompt metabolic evaluation and targeted therapy, helping prevent irreversible neuronal injury and metabolic decompensation.

This study has several limitations. The relatively small sample from two tertiary referral centers may limit generalizability and introduce referral bias. In addition, outcomes were assessed only at 3 months, which may not reflect long-term neurological and developmental sequelae. Larger multicenter studies with longer follow-up are needed to define prognostic factors, clarify genotype–phenotype correlations, evaluate early targeted therapies, and develop standardized diagnostic approaches for metabolic stroke.

## 5 Conclusion

Stroke secondary to inborn errors of metabolism (IEMs) should be considered in any child presenting with acute neurological deficits, particularly in the presence of consanguinity, a family history of similar episodes or unexplained deaths, associated systemic features, or neuroimaging findings beyond vascular territories. Our findings emphasize the broad clinical and genetic spectrum of IEM-associated stroke and highlight the importance of combining clinical assessment with neuroimaging, biochemical investigations, and molecular genetic testing to achieve an early diagnosis, guide targeted treatment, and facilitate genetic counselling and family screening.

## Data Availability

The datasets presented in this study can be found in online repositories. The names of the repository/repositories and accession number(s) can be found at: https://zenodo.org/records/21325187, doi: 10.5281/zenodo.21325187.
